# Role of Cannabinoids in Inflammation

**DOI:** 10.3390/molecules27020478

**Published:** 2022-01-12

**Authors:** Eric J. Downer

**Affiliations:** Discipline of Physiology, School of Medicine, Trinity Biomedical Sciences Institute, Trinity College Dublin, University of Dublin, D02 R590 Dublin, Ireland; edowner@tcd.ie

This Special Issue brings an update on some of the advances in research in the cannabinoid field, with focus on the impact of cannabinoids on inflammation. The issue brings together six articles, presenting some insights on novel cannabinoid targets in inflammation.

The contribution from Cardinal von Widdern et al. (2020) used an in vitro system to assess the proclivity of abnormal cannabidiol (abn-CBD) to modulate inflammation induced by lipopolysaccharide (LPS) in murine glial cells (astrocyte cultures and astrocyte-microglia co-cultures) [1]. Abn-CBD is a synthetic atypical cannabinoid, and using this compound, the authors demonstrate that abn-CBD (in the 1–10 μM range) ameliorated LPS-induced TNF-α and nitrite production in glial co-cultures from C57BL/6 wildtype mice. Similar effects were determined in CB_2_ knock-out astrocytic–microglia co-cultures. Abn-CBD significantly attenuated LPS-induced TNF-α expression in pure astrocytes. In addition, the ability of abn-CBD to reduce wound closure in glia using the scratch-wound assay was dependent on microglial cells. Interestingly, CB_2_ deficiency ameliorated the effect of LPS treatment on nitrite and TNF-α production in glia. These findings shed light on the mechanisms of action of abn-CBD in modulating glial reactivity and scar formation.

By focusing on the role of CB_2_ cannabinoid receptors in mediating the locomotor effects of the Khat extract, the research data from Geresu and colleagues (2019) have implications in terms of movement disorders linked to dysregulated dopamine signalling [2]. The leaves of *Catha edulis* Vahl. Endl., commonly known as khat, are used as an amphetamine stimulant, and the data presented in this article indicate that the khat extract, when delivered at 300 mg/kg to wildtype mice, increased locomotor activity. This increase was ameliorated by co-administration of JWH133 (5 mg/kg), indicating the involvement of the CB_2_ receptors in locomotor activity. Indeed, this was supported by evidence that the deletion of CB_2_ receptors on dopaminergic neurons exacerbated khat-induced hyperlocomotor activity. The authors also employed the use of 1-Methyl-4-phenyl-1,2,3,6-tetrahydropyridine (MPTP) to promote dopaminergic toxicity and motor deficits in mice, and khat and JWH133 reversed the impact of MPTP on motor deficits. In the final set of experiments, the authors indicate that khat has the proclivity to enhance tyrosine hydroxylase immunoreactivity in neurons in the ventral tegmental area in wildtype mice. This study adds to our understanding of the impact of cannabinoids and khat extracts on dopaminergic pathways and CNS movement disorders.

The third research article focuses on the role of endogenous signalling via the peroxisome proliferator-activated receptors (PPARs) in modulating fear and pain in Sprague Dawley rats [3]. Using antagonists for the three isoforms of PPARs (PPARα, PPARβ/δ, PPARγ), the authors demonstrate that systemic administration of PPARα and PPARβ/δ antagonists did not modulate fear-conditioned analgesia or formalin-evoked nociceptive behaviour, but both antagonists showed some propensity to prolong fear-related behaviour in formalin-injected animals. The PPARγ antagonist potentiated fear-related behaviour over the course of the trials. These findings suggest a role for PPAR signalling in conditioned fear in the presence of nociceptive tone. These findings have implications in terms of the endocannabinoid system, as studies elsewhere have shown that the *N*-acylethanolamines, anandamide, palmitoylethanolamide and oleolylethanolamide are endogenous ligands for PPARs, and their expression levels are elevated centrally in models of fear-conditioned analgesia [4].

The review article by Scheau et al. (2020) addresses the impact of phytocannabinoids, endocannabinoids and synthetic cannabinoid compounds on dermatological inflammatory disorders [5]. The review summarizes the complex pathological mechanisms driving skin inflammation and particularly focuses on the immune mechanisms contributing to skin disease. The authors comprehensively reviewed evidence of the role of cannabinoids in inflammatory events linked to psoriasis, allergic contact dermatitis, dermatomyositis and scleroderma. Particular attention is given to the therapeutic potential of cannabinoids in targeting signalling pathways contributing to skin tumorigenesis. Cannabinoids, or related compounds, may be administered transcutaneously (via patches, for example) in the treatment of various inflammatory skin disorders, minimizing unwanted systemic adverse effects and offering avenues for improving therapeutic drug delivery in dermatological inflammatory disorders.

Jones and Vlachou (2020) review the progress to date in terms of the therapeutic efficacy of both cannabidiol (CBD) and ∆^9^-tetrahydrocannabinol (THC) in multiple sclerosis (MS) [6]. This review is pertinent in terms of the clinical development of sativex^®^ (nabiximols). They comprehensively examine the impact of both CBD and THC in murine models of MS and suggest that the delivery of CBD and THC in combination is more effective at ameliorating the progression of disease in murine models of MS compared to the delivery of each phytocannabinoid alone in the disease model. The review also collates the body of research assessing the therapeutic effects of THC and CBD in human studies in people with (pw)MS. The frequencies of adverse events associated with cannabinoid administration in pwMS is summarized, and particular attention is given to the potential cognitive implications associated with the use of cannabinoids. The review concludes by presenting a convincing case in terms of the potential of sativex^®^ at effectively reducing neuropathic pain in pwMS.

The final review focuses specifically on the relationship between the endocannabinoid system and microglial cell activation, with focus on the contribution(s) of this relationship to neuroinflammation/neurodegeneration associated with Parkinson’s disease (PD) [7]. The review addresses the origin and function of microglia and covers the key role(s) played by microglia in neuroinflammation. There is abundant literature highlighting the contribution of microglial cell activation to the pathogenesis of PD; however, more research is needed to systematically decipher the contribution of microglial phenotypes to the stage of disease progression in PD. Microglia produce endocannabinoids and express components of the endocannabinoid system (cannabinoid receptors and endocannabinoid-metabolizing enzymes), the expression and production of which are dysregulated in neuroinflammatory disorders. The review collates the evidence demonstrating the proclivity of the cannabinoid system to modulate phagocytic and migratory activity in microglia, in addition to summarizing the effects of cannabinoids on cytokine production by microglial cells. The authors investigated the literature assessing the therapeutic role of cannabinoids by targeting microglial cell dysfunction and inflammation in PD, and conclude that further research is needed to address the impact of cannabinoids on further inflammatory models of PD, in addition to the need for clinical trial data on the efficacy of cannabinoids in PD.

In summary, the new manuscripts presented in this Special Issue demonstrate some key research angles of cannabinoid laboratories in the field of inflammation. Progress continues to be made to shed light on the propensity of exogenously administered cannabinoids, and the endocannabinoid system, to modulate inflammatory mechanisms contributing to the pathogenesis of disease.
